# Identifying Hidden Disability in Migraine Patients Using the Migraine Interictal Burden Scale-4

**DOI:** 10.7759/cureus.98195

**Published:** 2025-11-30

**Authors:** Norichika Hashimoto, Hatsuji Uno, Tetsurou Tsuji, Makoto Isozaki

**Affiliations:** 1 Department of Neurosurgery, University of Fukui, Fukui, JPN; 2 Department of Neurosurgery, Fukui General Hospital, Fukui, JPN

**Keywords:** cgrp antibody therapy, hit-6, interictal burden, mibs-4, patient-reported outcomes

## Abstract

Background

Migraine is increasingly recognized as a disorder with a substantial burden, not only during attacks but also during interictal periods. However, this dimension of disability remains under-assessed in routine clinical practice, partly because clinical attention has traditionally focused on acute attack symptoms, while patients themselves may perceive interictal periods as “normal” and therefore underreport the psychosocial impact. The Migraine Interictal Burden Scale-4 (MIBS-4) provides a concise measure of this hidden burden; however, its clinical relevance in Japanese outpatient populations has not been thoroughly investigated. This study aimed to evaluate the clinical utility of the MIBS-4 and identify its predictors in Japanese patients with migraine by examining its association with ictal disability (HIT-6) and other clinical variables.

Methods

We conducted a cross-sectional study involving 81 patients diagnosed with migraine at the neurosurgical outpatient clinic. Interictal burden was assessed using the MIBS-4, and ictal disability was evaluated using the Headache Impact Test-6 (HIT-6). Emotional distress was measured using validated screening tools. Multiple regression analyses were performed to identify predictors of MIBS-4 scores. The independent variables entered into the regression model were migraine subtype, monthly migraine days (MMD), disease duration, sex, age, and headache-related disability (HIT-6).

Results

Among 81 Japanese migraine patients, higher MIBS-4 scores were significantly associated with increased HIT-6 scores (β = 0.26, p < 0.001), indicating that greater headache-related disability contributes to an elevated interictal burden. Female sex was also associated with lower MIBS-4 scores (β = -1.49, p = 0.057), and longer disease duration showed a trend toward significance (p = 0.096). MMD, migraine subtype, and age were not significant predictors. Logistic regression revealed that higher HIT-6 scores (odds ratio (OR) = 1.33, p = 0.001) and longer disease duration (OR = 1.01, p = 0.016) were significantly associated with a clinically high interictal burden (MIBS-4 ≥5).

Conclusion

In this study, MIBS-4 scores were shown to be associated with HIT-6 scores and disease duration, but not with monthly migraine days. These findings support the utility of MIBS-4 in capturing interictal disability independently of attack frequency. Routine use of MIBS-4 in outpatient practice may help uncover hidden disease burden and guide preventive treatment strategies, particularly for patients with a long disease duration or elevated headache-related disability.

## Introduction

Migraine imposes a persistent burden not only during attacks but also between them. This interictal disability, including difficulties in maintaining work productivity, social participation, or family responsibilities, remains under-recognized in clinical practice [1,2]. Such a hidden burden contributes to reduced quality of life and may lead to inadequate treatment strategies.

Despite its clinical relevance, interictal disability has been less frequently assessed compared to ictal burden. The Migraine Interictal Burden Scale-4 (MIBS-4) was recently developed as a validated tool to evaluate between-attack disability [3,4]. Recent data from the OVERCOME (Japan) study highlight its importance: among 17,071 respondents, 41.5% reported moderate-to-severe interictal disability, with notable impacts on sleep, mood, work productivity, and social functioning, even among patients with low-frequency episodic migraine [5,6].

Although international studies have demonstrated the utility of the MIBS-4, evidence in Japanese clinical settings remains limited. Clarifying the predictors of interictal burden may help identify patients who require preventive strategies beyond attack frequency.

The objective of this study is to evaluate the clinical utility of the Migraine Interictal Burden Scale-4 (MIBS-4) and to identify its predictors in Japanese patients with migraine. This was achieved by examining its association with ictal disability (Headache Impact Test-6, HIT-6) and other clinical variables [7].

## Materials and methods

Study design and participants

This cross-sectional observational study was conducted at the outpatient neurosurgery department of Fukui General Hospital, Fukui, Japan, between September 2024 and August 2025. A total of 81 adult patients diagnosed with migraine were enrolled in this study. The inclusion criteria required participants to be over 17 years of age; meet the diagnostic criteria for migraine according to the International Classification of Headache Disorders, 3rd edition (ICHD-3); and be capable of completing self-administered questionnaires. Patients with comorbid neurological disorders and incomplete data were excluded.

This study was exploratory in nature; therefore, a formal sample size calculation was not conducted. To minimize the risk of selection bias, consecutive patients who met the inclusion criteria were enrolled in the neurosurgery outpatient department of Fukui General Hospital.

Migraine subtypes were classified based on the ICHD-3 definitions. Episodic migraine was defined as fewer than 15 headache days per month, whereas chronic migraine was defined as 15 or more headache days per month, including at least eight days with migraine features.

Assessment tools

Two validated instruments were used to evaluate the impact of migraine on daily life. The Japanese version of the MIBS-4 assesses the burden during interictal periods in four domains: family, social, work/school, and emotional impact. Each item is scored from 0-3, yielding a total score ranging from 0-12. Scores of 0-4 indicate a low burden, while scores ≥5 indicate a high interictal burden. This cutoff has been validated in previous studies and is widely used to define clinically significant interictal burden, justifying its use as an outcome measure in this study [4,8].

In clinical migraine assessment, emphasis has traditionally been placed on the attack frequency and severity, particularly monthly migraine days (MMD). While tools such as the Migraine Disability Assessment (MIDAS) and HIT-6 effectively quantify ictal burden, they do not capture the limitations experienced between the attacks. This interictal burden, which may affect social, emotional, and occupational functioning, is often under-recognized in routine care settings. Psychometric evaluation has demonstrated good internal consistency (Cronbach’s α ≈0.80) and convergent validity with other headache-related measures, including the HIT-6. The Japanese version used in this study was developed under the supervision of Eli Lilly, Japan, with editorial oversight from Professor Takao Takeshima [9]. According to a correspondence with Eli Lilly, Japan, no formal license is required for academic use of this software. The original publication by Lipton et al. was cited as a reference in this study [4,8].

The Japanese version of HIT-6 measures headache-related impact on frequency, social functioning, cognitive functioning, and psychological distress, with scores ranging from 36 to 78. The clinical interpretation of score ranges is as follows: 36-49 = little/no impact, 50-55 = moderate impact, 56-59 = substantial impact, and ≥60 = severe impact. Psychometric studies have confirmed acceptable internal consistency (Cronbach’s α ≈0.82-0.92), good test-retest reliability, and validity in migraine populations [10]. The use of the Japanese version of HIT-6 was formally requested and approved by Eli Lilly, Japan. The license number is mol202412310023, and permission was granted by Medical Tribune, Inc. for reproduction from Rinsho Iyaku: Journal of Clinical Therapeutics & Medicine.

Ethical approval

The study protocol was approved by the Institutional Review Board of Fukui General Hospital (approval no. 2025-23). Written informed consent was obtained from all the participants. This study was conducted in accordance with the Declaration of Helsinki and relevant national ethical guidelines. Participant confidentiality and anonymity were strictly maintained, and the data were used solely for academic purposes. No financial or non-financial conflicts of interest influenced the study design, conduct, or reporting of this study.

Statistical analysis

Statistical analyses included descriptive statistics (mean, standard deviation, median, and interquartile range). Skewed variables were summarized using the median and interquartile range (IQR) to appropriately represent their distributions. Binary logistic regression was used to identify the predictors of a high interictal burden, defined as MIBS-4 ≥5. The independent variables considered included HIT-6 scores, sex, migraine type, MMD, age, and disease duration. All clinically relevant variables were included in multivariable models. All analyses were performed using EZR version 1.55 (based on R version 4.2.2; Saitama Medical Center, Jichi Medical University, Saitama, Japan) and GraphPad Prism 5 (GraphPad Software, San Diego, CA), with statistical significance set at p < 0.05. No missing data were found in this study; therefore, no imputation or exclusion procedures were required.

## Results

Participant characteristics

A total of 81 patients with migraine were included in the study. The median age was 46.0 years (IQR: 25.5-52.5), and 64 patients　(79.0%) were female. Chronic migraine was diagnosed in 27 patients (34.2%).

The average of migraine history (years) was 10.27± 9.02 (IQR: 3.0-19.0), and MMD was 10.00 ± 7.02. The mean HIT-6 score was 61.2 ± 7.23, and the mean MIBS-4 score was 2.67 ± 3.29. Migraine history was 7.0 years (IQR: 3.0-19.0), and MMD was 9.0 (IQR: 4.0-15.0). The mean HIT-6 score was 61.2 ± 7.23, and the MIBS-4 score was 1.0 (IQR: 0-5.0). Overall, 25.9% of patients exhibited a high interictal burden (MIBS-4 ≥5).

Preventive treatments included conventional medications in 53 patients (65.4%) and calcitonin gene-related therapies in 34 patients 41.9%). Some patients received both conventional and calcitonin gene-related peptide (CGRP)-targeted preventive treatments; for acute treatment, 56 patients (69.1%) used triptans, and 38 patients (46.9%) used nonsteroidal anti-inflammatory drugs (NSAIDs).

The baseline characteristics are summarized in Table 1.

**Table 1:** Demographic and Clinical Characteristics of Japanese Migraine Patients (N = 81) Summary of age, sex distribution, disease duration, monthly migraine days (MMD), migraine type, acute and preventive treatment use, and impact scores (HIT-6 and MIBS-4). Data are presented as mean ± standard deviation, median (interquartile range), and range or percentage as appropriate. Abbreviations: MMD, Monthly Migraine Days; HIT-6, Headache Impact Test-6; MIBS-4, Migraine Interictal Burden Scale-4; NSAIDs, Non-Steroidal Anti-Inflammatory Drugs; CGRP, Calcitonin Gene-Related Peptide

Category	Variable	Summary measure	n (%)
Demographics	Age (years)	46.0 (25.5–52.5)	
	Female (%)	—	64 (79.0%)
Headache Burden	Migraine History (years)	7.0 (3.0–19.0)	
	Monthly Migraine Days (MMD)	9.0 (4.0–15.0)	
	Migraine Type (Chronic Migraine, %)	—	27 (33.3%)
Treatment Use	Use of Acute Triptans	—	56 (69.1%)
	Use of Acute NSAIDs	—	38 (46.9%)
	Use of Preventive Conventional Drugs	—	53 (65.4%)
	Use of CGRP Preventive Drugs	—	34 (41.9%)
Impact Scores	HIT-6 Score	61.2 ± 7.23	
	MIBS-4 Score	1.0 (0.0–5.0)	

Association between MIBS-4 and HIT-6 scores

**Figure 1:**
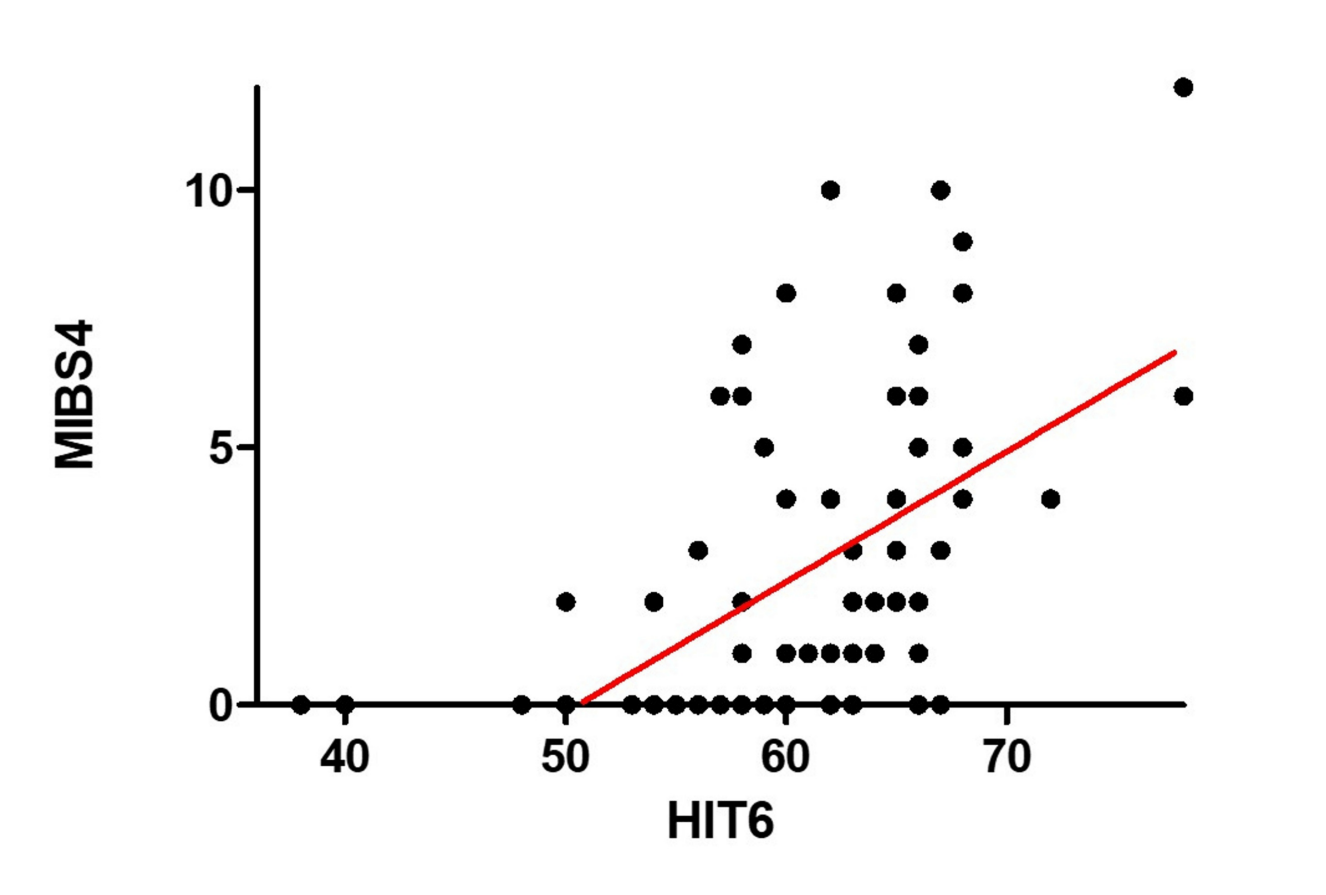
Correlation Between HIT-6 and MIBS-4 Scores Scatter plot illustrating the relationship between HIT-6 and MIBS-4 scores among Japanese patients with migraine. A statistically significant positive correlation was observed (r² = 0.3094, p < 0.0001), although the explanatory power was modest. Abbreviations: HIT-6, Headache Impact Test-6; MIBS-4, Migraine Interictal Burden Scale-4

Simple linear regression showed a significant positive association between the MIBS-4 and HIT-6 scores (slope = 0.2531 ± 0.043, R² = 0.309, p < 0.0001). Higher MIBS-4 scores were associated with a greater headache-related impact. The model explained 30.9% of the variance in the MIBS-4 scores. This relationship is illustrated in Figure 1, which presents a scatter plot of the HIT-6 and MIBS-4 scores among Japanese migraine patients.

Multiple linear regression predicting MIBS-4 scores in migraine patients

A multiple linear regression analysis was used to identify predictors of interictal burden (MIBS-4). The HIT-6 score was significantly associated with a higher MIBS-4 score (β = 0.26, 95% CI: 0.102-0.278, p < 0.001), indicating that greater headache-related disability contributes to an increased burden. Female sex was also associated with lower scores (β = -1.49, 95% CI: -3.13 to -0.25, p = 0.057).

Disease duration was not significantly (p = 0.096), whereas migraine type, MMD, and age were not significant. The model explained 28.0% of the variance (R² = 0.280; adjusted R² = 0.218; p = 0.0007) (Table 2).

**Table 2:** Multiple Linear Regression Predicting MIBS-4 Scores Results of a multiple linear regression model examining predictors of interictal burden (MIBS-4 scores) in Japanese migraine patients (N = 81). Independent variables included HIT-6 scores, sex, migraine type, MMD, age, and disease duration. Significant predictors were HIT-6 (β = 0.26, 95% CI: 0.102–0.278, p < 0.001). Other variables were not statistically significant. Abbreviations: HIT-6, Headache Impact Test-6; MIBS-4, Migraine Interictal Burden Scale-4; MMD, Monthly Migraine Days; CI, Confidence Interval

Predictor	Estimate (β)	95% Confidence Interval	P-value	Significance	Clinical Interpretation
HIT-6	0.26	0.102 to 0.278	<0.001	***	Greater headache impact during attacks is strongly associated with higher interictal burden.
Sex (Female = 1)	–1.49	–3.13 to –0.25	0.057	† (trend)	Female patients tend to report lower interictal burden.
Migraine Type	0.003	–1.70 to 2.48	0.997	n.s.	No significant difference between chronic and episodic migraine in terms of interictal burden.
Monthly Migraine Days	–0.006	–0.16 to 0.13	0.939	n.s.	Attack frequency alone does not significantly predict interictal burden.
Age	–0.018	–0.064 to 0.015	0.405	n.s.	Slight trend toward lower burden with age, possibly due to improved coping strategies.
Disease Duration	0.068	–0.008 to 0.142	0.096	† (trend)	Longer disease duration may be associated with increased cumulative burden.

Predictors of high interictal burden (MIBS-4 ≥5)

To identify predictors of clinically significant interictal burden, binary logistic regression analysis was performed using MIBS-4 ≥5 as the outcome. The model included the HIT-6 score, disease duration, sex, migraine type, MMD, and age.

Higher HIT-6 scores were significantly associated with increased odds of a high interictal burden (OR = 1.33, 95% CI: 1.11-1.60, p = 0.002), indicating that greater headache-related disability contributes to an elevated burden. Longer disease duration was also significantly associated with the significant association (OR 1.11, 95% CI: 1.02-1.21, p = 0.016).

Female sex was associated with a higher burden, although not statistically significant (OR = 0.25, 95% CI: 0.052-1.15, p = 0.152). Migraine type, MMD, and age were not significant predictors of headache frequency. The full results are presented in Table 3.

**Table 3:** Predictors of High Interictal Burden (MIBS-4 ≥ 5) in Migraine Patients Binary logistic regression analysis identifying predictors of high interictal burden (defined as MIBS-4 ≥ 5) in Japanese migraine patients (N = 81). Higher HIT-6 scores and longer disease duration were significantly associated with increased odds of high interictal burden. Female sex showed a trend-level association. Migraine type, monthly migraine days (MMD), and age were not significant predictors. Abbreviations: HIT-6, Headache Impact Test-6; MIBS-4, Migraine Interictal Burden Scale-4; MMD, Monthly Migraine Days; OR, Odds Ratio; CI, Confidence Interval.

Predictor	Odds Ratio (OR)	95% Confidence Interval	P-value	Significance	Clinical Interpretation
HIT-6 Score	1.33	1.11-1.60	0.001	**	Greater headache impact increases odds of high interictal burden.
Disease Duration (years)	1.01	1.02-1.21	0.016	*	Longer disease duration is associated with greater interictal burden.
Sex (Female = 1)	0.25	0.052-1.15	0.152	n.s.	Female sex may be associated with higher burden, though not statistically significant.
Migraine Type (Chronic)	1.57	0.18-14.70	0.678	n.s.	No significant difference between chronic and episodic migraine.
Monthly Migraine Days	1.01	0.85-1.18	0.908	n.s.	Attack frequency does not predict interictal burden.
Age	0.98	0.94-1.02	0.395	n.s.	No significant association with age.

## Discussion

In this study, we found that higher HIT-6 scores were significantly associated with greater interictal burden (MIBS-4), while disease duration and female sex showed no statistically significant associations. These findings highlight the central role of headache-related disability in predicting interictal burden among migraine patients. MIBS-4 scores were not significantly correlated with MMD, supporting their role as independent measures of disease impact. In this cohort, 25.9% of patients exhibited a high interictal burden (MIBS-4 ≥5), indicating that disability beyond the attack phase is common.

These findings are consistent with prior international studies. In a cross-sectional survey of migraine patients in the United States and Germany, Hubig et al. reported that 67% of participants exhibited severe interictal burden (MIBS-4 ≥5), with significant associations between MIBS-4 scores and depression, CGRP monoclonal antibody treatment, and HIT-6 scores [3,8]. Similarly, Lo et al. conducted qualitative interviews in the US, UK, and Canada, revealing that patients experienced substantial emotional and functional disruptions between migraine episodes, including anxiety, social withdrawal, and impaired productivity [9]. These studies underscore the multidimensional nature of interictal burden and support the utility of the MIBS-4 in capturing disease impact beyond attack frequency.

Possible explanations for the lower proportion observed in our cohort include differences in study setting, sample characteristics, and cultural background. Specifically, the survey was self-administered, and some patients may have under-recognized their own anxiety or loss of vitality during the interictal period. In addition, cultural factors unique to Japan, and particularly to Fukui Prefecture, may have influenced responses. Fukui has the highest rate of dual-income households in Japan, and a cultural tendency toward endurance and perseverance may contribute to underreporting of interictal burden. These contextual factors should be considered when interpreting the relatively lower prevalence compared to international studies.

Regression analyses identified the HIT-6 score as a significant predictor of higher MIBS-4 scores, whereas disease duration showed a trend toward significance, and female sex was not significantly associated. These results are consistent with those of Lampl et al., who reported that interictal burden is influenced by psychosocial and demographic factors [11].

Limitations

This study has several limitations. It was conducted in a single-center setting with a relatively small sample size, and its cross-sectional design does not allow causal inference. In addition, the use of self-administered questionnaires may have introduced reporting bias. Furthermore, although consecutive enrollment was performed to minimize the risk of selection bias, the possibility of residual bias cannot be excluded. The study did not include certain potentially relevant variables, such as socioeconomic status, education level, or comorbidities other than neurological disorders, which may have influenced the outcomes. Unmeasured confounders, including psychosocial and lifestyle factors, may have also affected the results. Moreover, the single-center design limits the generalizability of our findings to a broader population.

Strengths and contributions

Despite these limitations, our study contributes to the existing evidence by applying the MIBS-4 to a general neurosurgical outpatient population and examining its relationship with the HIT-6 and clinical variables.

Future directions

Further prospective studies are warranted to determine whether treatment strategies guided by interictal burden, such as the early initiation of CGRP-targeted therapy, can improve long-term outcomes in migraine care.

## Conclusions

The MIBS-4 effectively captures interictal disability independent of attack frequency. Elevated MIBS-4 scores were associated with higher HIT-6 scores and longer disease duration, identifying a clinically relevant subgroup - particularly those with longer disease duration and elevated headache-related disability - that may benefit from preventive treatment. Given its simplicity and suitability for outpatient use, the MIBS-4 complements ictal assessments and supports a more comprehensive approach to migraine treatment. Future prospective studies are needed to evaluate whether treatment strategies guided by interictal burden can improve long-term outcomes in migraine management.

## References

[1] Vincent M, Viktrup L, Nicholson RA, Ossipov MH, Vargas BB (2022). The not so hidden impact of interictal burden in migraine: a narrative review. Front Neurol.

[2] Renjith V, Pai MS, Castelino F, Pai A, George A (2016). Clinical profile and functional disability of patients with migraine. J Neurosci Rural Pract.

[3] Hubig LT, Smith T, Williams E (2022). Measuring interictal burden among people affected by migraine: a descriptive survey study. J Headache Pain.

[4] Buse DC, Rupnow MF, Lipton RB (2009). Assessing and managing all aspects of migraine: migraine attacks, migraine-related functional impairment, common comorbidities, and quality of life. Mayo Clin Proc.

[5] Matsumori Y, Ueda K, Komori M (2022). Burden of migraine in Japan: results of the observational survey of the epidemiology, treatment, and care of migraine (OVERCOME [Japan]) study. Neurol Ther.

[6] Awaki E, Takeshima T, Matsumori Y (2024). Impact of migraine on daily life: results of the observational survey of the epidemiology, treatment, and care of migraine (OVERCOME [Japan]) study. Neurol Ther.

[7] Ooba S, Ooba H (2024). [The evaluation of each migraine treatment using migraine interictal burden scale-4 (MIBS-4)]. Japanese Journal of Headache.

[8] Stewart WF, Lipton RB, Dowson AJ, Sawyer J (2001). Development and testing of the migraine disability assessment (MIDAS) questionnaire to assess headache-related disability. Neurology.

[9] Lo SH, Gallop K, Smith T (2022). Real-world experience of interictal burden and treatment in migraine: a qualitative interview study. J Headache Pain.

[10] Rendas-Baum R, Yang M, Varon SF, Bloudek LM, DeGryse RE, Kosinski M (2014). Validation of the headache impact test (HIT-6) in patients with chronic migraine. Health Qual Life Outcomes.

[11] Lampl C, Seng E, Vincent M (2024). Interictal burden in migraine patients at the outset of CGRP monoclonal antibody prevention. J Headache Pain.

